# Counterfactual protocol within device independent framework and its insecurity

**DOI:** 10.1038/s41598-020-62812-3

**Published:** 2020-04-03

**Authors:** Suhaili Kamaruddin, Jesni Shamsul Shaari, Piotr Kolenderski

**Affiliations:** 1 Faculty of Science, International Islamic University Malaysia (IIUM), Jalan Sultan Ahmad Shah, Bandar Indera Mahkota, 25200 Kuantan, Pahang Malaysia; 2 Faculty of Physics, Astronomy and Informatics, Nicolaus Copernicus University, Grudziadzka 5, 87-100 Toruń, Poland

**Keywords:** Quantum information, Single photons and quantum effects

## Abstract

We consider the counterfactual protocol proposed in *Phys. Rev. Lett*., **103**, 230501 (2009) within a device independent framework and show how its security can easily be compromised. Capitalising on the fact that the protocol is based on the use of a single photon entanglement phenomenon, we propose an equivalent protocol. It can be made secure within such a pessimistic framework against a supra-quantum Eve limited only by the no-signalling principle. The equivalence the protocol demonstrates the possibility of device independent framework for counterfactual quantum cryptography.

## Introduction

Quantum key distribution (QKD) scheme allows a secret key to be shared between two parties, say Alice and Bob, by transmitting information carrier through the quantum channel^1^. However, Noh’s protocol^2^, in which we will refer to as counterfactual QKD (CQKD), had proposed that a secret key can still be shared by Alice and Bob without any qubit travelling between them by virtue of counterfactual phenomenon. This phenomenon enables the authorised parties to infer the presence of an object effectively without having to measure it^2^.

In order for this phenomenon to take effect, one requires as a resource, a single photon entanglement which can be attained by submitting a single photon to a beam splitter. The single photon entanglement refers to a phenomenon of entanglement between the photon numbers in two spatially separated modes where one mode is connected to Bob as the quantum channel while the other remains with Alice^3^. Given a 50:50 beam splitter, photons can be found half of the time on the quantum channel.

Unlike the conventional QKD scheme relying on transmission of signals, the CQKD protocol presents a security advantage where Eve cannot fully access the qubits. A simple example how such a feature can be useful is in cases where multiphoton signals are used and Eve cannot determine the photon number without having access to the mode in Alice’s site^2^. The security of CQKD protocol has been proved using an ideal single photon source^4,5^ and weak coherent states^6^.

Existing security analysis of most QKD scheme assume that the measurement devices are trusted and that the authorised parties have perfect control of the photon source^7–10^. Nevertheless, in a device independent setting this is not necessarily true as the most pessimistic scenario assumes the possibility that the devices could have been fabricated by a malicious Eve with the legitimate parties being ignorant of the flaws therein. Conventionally, this forces the devices to be seen as black boxes.

In the following, we will show that given the device independent framework for secrecy, the CQKD protocol^2^, and the equivalent protocol described in ref. ^11^, is in fact completely insecure. Though actually this is the case even if Eve is limited only by quantum physics. We propose a setup considered as a set of black boxes for Alice and Bob, which can completely simulate the expected statistics of the CQKD while allowing Eve to have full knowledge of the shared key. In the first strategy, the setup consist of entangled sources as signals while the second is separable system. We then identify the essential source of insecurity and propose a modification and subsequently a proper CQKD, which would be secure within a device independent framework.

## CQKD within Device Independent Framework

Let us begin with a review of the CQKD framework^2^, which security we analyse here. It must be noted that this description is completely equivalent to the one proposed by ref. ^2^; though it has, to a certain extent, some simplicity in its description.

**Figure 1:**
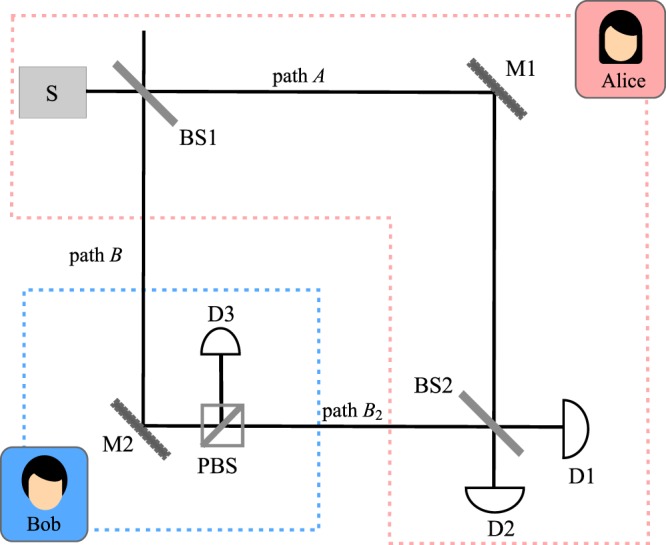
A diagram of CQKD proposed by ref. ^11^. BS1 and BS2 are beam splitters, D1, D2 and D3 are detectors, M1 and M2 are mirrors and PBS is a polarising beam splitter. Path *A*, *B*_1_ and *B*_2_ are the paths from the source towards M1, from the beam splitter BS1 to the mirror M2 and from M2 to BS2, respectively.

We imagine that two parties, say Alice and Bob, share a setup as depicted in Fig. 1. The protocol starts when Alice triggers the photon source (S) that emits a pulse containing a single-photon. Depending on Alice’s random choices, the single-photon could be in either horizontally polarised state $| H\rangle$ which represent Alice’s bit ‘0’ or vertically polarised state $| V\rangle$ as bit ‘1’. The single-photon pulse passes through a 50:50 beam splitter (BS1) in which the output results in the following state (in accordance with Alice’s choice of polarisation state):

1 $${| \Psi \rangle }_{H}=\frac{1}{\sqrt{2}}({| H\rangle }_{A}{| 0\rangle }_{B}-{| 0\rangle }_{A}{| H\rangle }_{B})$$

2 $${| \Psi \rangle }_{V}=\frac{1}{\sqrt{2}}({| V\rangle }_{A}{| 0\rangle }_{B}-{| 0\rangle }_{A}{| V\rangle }_{B})$$

where ${| 0\rangle }_{i}$ denotes the vacuum state with *i* ∈ *A*, *B* represent the path towards Alice’s mirror M1 and Bob’s site, respectively. We further denote the paths *A*, *B*_1_ and *B*_2_ for the paths from the source towards M1, from the beam splitter BS1 to the mirror M2 and from M2 to BS2 respectively.

The pulse that travels through path *B* is reflected by M2 before entering the input port of the polarising beam splitter (PBS) on Bob’s site. Bob will randomly choose between horizontal and vertical polarisation to represent his bit. The PBS is configured such that, if Bob’s choice of polarisation is not equal to Alice, the PBS will transmit the pulse towards BS2 and the split pulse that travels in the two modes are recombined at beam splitter, BS2. In an ideal setting, the interference effect will cause the photon to be detected at D1 with certainty.

However, if the incoming polarisation is the same with Bob’s choice, the pulse will be reflected towards Bob’s measurement setting which consists of photon detector, D3. The measurement process will cause the state ${| \Psi \rangle }_{H}$ to collapse to either ${| H\rangle }_{A}{| 0\rangle }_{B}$ or ${| 0\rangle }_{A}{| H\rangle }_{B}$; or state ${| \Psi \rangle }_{V}$ to either ${| V\rangle }_{A}{| 0\rangle }_{B}$ or ${| 0\rangle }_{A}{| V\rangle }_{B}$, which eventually destroys the interference. In the event that the state collapses to either ${| H\rangle }_{A}{| 0\rangle }_{B}$ or ${| V\rangle }_{A}{| 0\rangle }_{B}$, the detector D1 and D2 in Alice’s site will click with equal probability.

On the other hand, if the state collapses to either ${| 0\rangle }_{A}{| H\rangle }_{B}$ or ${| 0\rangle }_{A}{| V\rangle }_{B}$, the detector D3 will click with certainty. At the end of transmission, Alice and Bob will reveal which of their detectors click. The case of detector D3 clicking implies that Alice gets nothing, while a click of either D1 or D2 implies that Bob effectively did not receive a photon. As D1 also clicks in the case of an interference, only the click at D2 provides Alice with a conclusive guess of Bob’s choice of polarisation. Thus the raw key will be extracted from the event in which detector D2 clicks.

## Security Analysis

In this section, we describe the CQKD within a device independent scenario in which Alice and Bob are provided with untrusted devices and they have no knowledge of the internal function of the QKD devices. The adversary may configure the devices such that they simulate the results that would be obtained from executing a counterfactual QKD protocol as described above.

**Figure 2:**
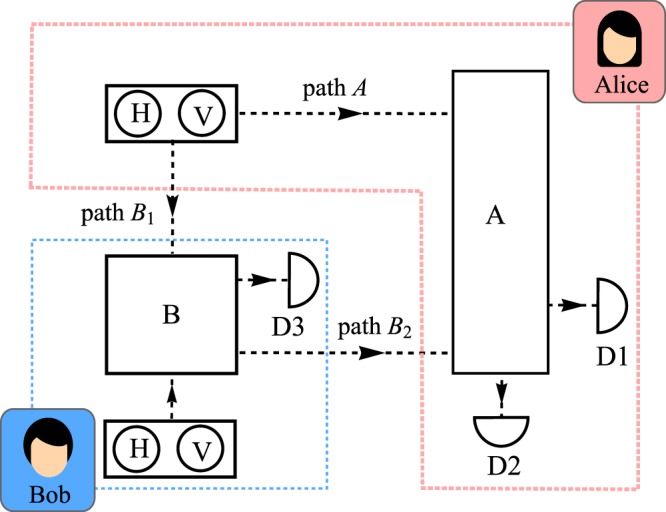
A proposed diagram of CQKD within device independent scenario. Box ‘A’ and ‘B’ represent the measurement devices for Alice and Bob, respectively. D*i* are the detectors. The button ‘H’ and ‘V’ represent the horizontal and vertical polarisation input.

In what follows, we can view these devices as black boxes (‘A’ for Alice and ‘B’ for Bob) each provided with binary input, say a ‘H’ and a ‘V’ button as potrayed in Fig. 2. For definiteness, we define ‘H’ as bit ‘0’ and ‘V’ as bit ‘1’. Path *A* and *B*_1_ are the paths from the input buttons in Alice’s site towards M1 and from Alice’s input buttons to Box B, respectively. Meanwhile, path *B*_2_ is the path connecting Box B and Box A. We further consider two different strategies by Eve in determining how the black boxes should behave. In either case, Eve would be using tripartite states distributing one subsystem to Alice and one to Bob. In the first strategy, an entangled bipartite state of Alice and Bob is separable from a (relevant) third parity state. In the second approach, the tripartite states are completely separable. We will now investigate the two strategies.

### CQKD with entangled states

The requirement to disclose ‘which detector clicked’ in public channel^2,11^ was intended to allow Alice and Bob to know when a bit is accepted for key generation purpose. However, this provides Eve with information on the bit string regardless of whether the qubits are entangled or not.

Let us now propose a protocol by prescribing requirements of how the boxes should behave in order to replicate the effects of the counterfactual QKD. Suppose that the states, ${| \Psi \rangle }_{m}$ being distributed are given by:

3 $${| \Psi \rangle }_{m}=\frac{({| 1\rangle }_{A}{| 0\rangle }_{B}-{| 0\rangle }_{A}{| 1\rangle }_{B})}{\sqrt{2}}\otimes {| m\rangle }_{B},$$

where *m* ∈ {*H*, *V*} depends on Alice’s choice of a button. Since the state ${| H\rangle }_{B}$ and ${| V\rangle }_{B}$ can be measured perfectly without disturbing the entangled state, then knowing ‘which detector clicked’ will allow Eve to know with certainty which bit is accepted as a key. Hence, the revelation of ‘which detector clicked’ as proposed by refs. ^2,11^ has become the main loophole in this protocol as Eve knows precisely well on the values of *m*, which she can determine.

One way of closing this loophole while still allowing for the legitimate parties to share a key is by having Alice to only declare when detector D2 clicks. In this way, whenever Bob does not measure a photon (D3), he would know when D2 clicks thus not use those for key sharing and when D1 clicks for key bits.

The second less obvious loophole is the case for Bob’s resending of a qubit in the path *B*_2_. Hence, if Alice and Bob were to drop this requirement i.e. they do not reveal which detector clicked in the public channel, or at most mention only when D2 clicks, and assure themselves that their first two qubits are in fact a maximally entangled states (which can violate a Bell inequality) then it is possible for them to extract a secure key.

### CQKD with separable states

Now, suppose the state that is really being distributed is a three-qubit state, either:

4 $${| 0\rangle }_{A}{| 1\rangle }_{B}{| m\rangle }_{B}\quad \,{\rm{o}}{\rm{r}}\,\quad {| 1\rangle }_{A}{| 0\rangle }_{B}{| m\rangle }_{B}$$

where *m* ∈ {*H*, *V*} depends on Alice’s choice of a button and the subscript *A* and *B* represent the qubit that is being distributed to Alice and Bob, respectively. While we do not make any requirement on state ${| m\rangle }_{B}$ to be a polarised state we nevertheless assume so in what follows for the sake of simplicity. The two-qubit state (either ${| 1\rangle }_{B}{| m\rangle }_{B}$ or ${| 0\rangle }_{B}{| m\rangle }_{B}$) would then be sent to Bob’s box, B. Bob will also randomly choose between his ‘H’ or ‘V’ button.

At first glance, it may seems as if information is leaked out of Alice’s site by sending the state ${| m\rangle }_{B}$ over to Bob. However, in a counterfactual perspective, it is crucial that the state ${| m\rangle }_{B}$ i.e the polarisation degree of freedom to be accessible to Bob otherwise the PBS cannot work. Eve would eventually know the values of *m* as she can make a measurement to distinguish the two polarisation states perfectly. Based on the choices made by Alice and Bob, we will consider the following two cases.

- **Case 1: Alice’s and Bob’s bit do not match**. Bob’s device will resend the second qubit to Alice’s site. This qubit along with her qubit would be inputs to box A in which would result in D1 clicking. This replicates the interference effect of the counterfactual QKD. We note that while this may seemingly ‘violate’ a requirement of device-independence where no information is leaked from Bob’s station, we argue this to be exceptional given the necessary channel (path *B*_2_) from Bob to Alice in a counterfactual setup.
- **Case 2: Alice’s and Bob’s bit coincide**. Bob’s box will not send anything towards Alice’s site. This action is similar to the path-blocking procedure as in refs. ^2,11^. We then consider the following scenarios:

1. In the event where Eve had distributed ${| 1\rangle }_{A}{| 0\rangle }_{B}{| m\rangle }_{B}$, Alice’s qubit ${| 1\rangle }_{A}$ will be submitted to box A to result in either detector D1 or D2 clicking with equal probability.
2. On the other hand, had Eve distributed ${| 0\rangle }_{A}{| 1\rangle }_{B}{| m\rangle }_{B}$, then Bob’s detector D3 will click. When box A detects Alice’s qubit as ${| 0\rangle }_{A}$, neither D1 nor D2 click.

The above can be achieved by first equipping box B with a measurement device to distinguish between the polarisation states of the third incoming qubit, ${| m\rangle }_{B}$ in order to measure whether it is horizontally or vertically polarised. Since it is orthogonal, then it can be done perfectly. We further require box B to act as follows: when Bob inputs a choice for polarisation (using either the H or V button), his choice would be compared to the polarisation of the third incoming qubit. If they are the same, a further measurement is made to distinguish between states ${| 0\rangle }_{B}$ and ${| 1\rangle }_{B}$ of the second qubit. In the case of the latter, the detector D3 is fired. Either way the process for box B ends and no qubit is sent out of Bob’s site. On the other hand, if the polarisation of the incoming qubit is different from Bob’s button choice, the second qubit is sent to Alice.

In order to simulate the counterfactual protocol we propose the following ansatz. For Case 1, we require that box A to behave as such that the probability of detector D*j* clicking given ${| i\oplus 1\rangle }_{B}{| i\rangle }_{A}$ is written as

5 $$P({\rm{D}}j{| | i\oplus 1\rangle }_{B}{| i\rangle }_{A})=\frac{1+{(-1)}^{j+1}}{2},$$

in which *j* = 1, 2 and *i* = 0, 1. Meanwhile, in Case 2 we need box A to behave as such that the probability of detector

6 $$P({\rm{D}}j{| | {\rm{n}}{\rm{o}}{\rm{i}}{\rm{n}}{\rm{p}}{\rm{u}}{\rm{t}}\rangle }_{B}{| i\rangle }_{A})=\left\{\begin{array}{ll}\frac{1}{2}, & i=1\\ 0, & i=0\end{array}\right.$$

for *j* = 1, 2 with ${| \mathrm{no}\mathrm{input}\rangle }_{B}$ represents the event when there is no incoming qubit from Bob. This box can be done by virtue of having the controlled-NOT gate called *CNOT*, which is defined as $CNOT:| b,a\rangle \to | b,a\oplus b\rangle$. Hence, let us reconsider both cases.

In Case 1, box A will receive the second qubit from Bob as well as Alice’s qubit as inputs. Box A will then perform the *CNOT* function on either one of the following:

7 $$CNOT{| 0\rangle }_{B}{| 1\rangle }_{A}$$

8 $$CNOT{| 1\rangle }_{B}{| 0\rangle }_{A}$$

with Alice’s resulting state would eventually be detected by either detector D1 or D2. Assuming that detector D1 will detect state ${| 0\rangle }_{A}$ and D2 will detect state ${| 1\rangle }_{A}$, the above *CNOT* function will eventually result in D2 only clicking.

When Alice’s and Bob’s bit are the same, no qubit from Bob will be sent out to box A. In the event where box A detects Alice’s qubit as ${| 1\rangle }_{A}$, it will perform a *CNOT* function on state $| x+\rangle =(| 0\rangle +| 1\rangle )$/$\sqrt{2}$ along with Alice’s state written as

9 $$CNOT| x+\rangle {| 1\rangle }_{A}.$$

We can assume that the state $| x+\rangle$ is supplied by the box A. As a result, with equiprobability detector D1 and D2 will click. On the other hand, if state ${| 0\rangle }_{A}$ is being detected, then box A will end its process.

As demonstrated above, Eve can perfectly simulate the protocol by distributing a system that is made up of entirely separable states. As she knows the values of *m*, as well as when Alice and Bob accepts or rejects a run, Eve basically has complete knowledge of the key. It is then obvious that the protocol presented by refs. ^2,11^ are not secure in a device independent context.

By not revealing the information on ‘which detector clicked’, Eve would not have known which bit is going to be accepted even if the strings for raw key is publicly broadcasted. It would seem that both scenarios can be viewed as a separate system. In what follows, we are going to propose a framework for device independent CQKD (DI-CQKD) based on these conditions.

## The Proposed DI-CQKD

**Figure 3:**
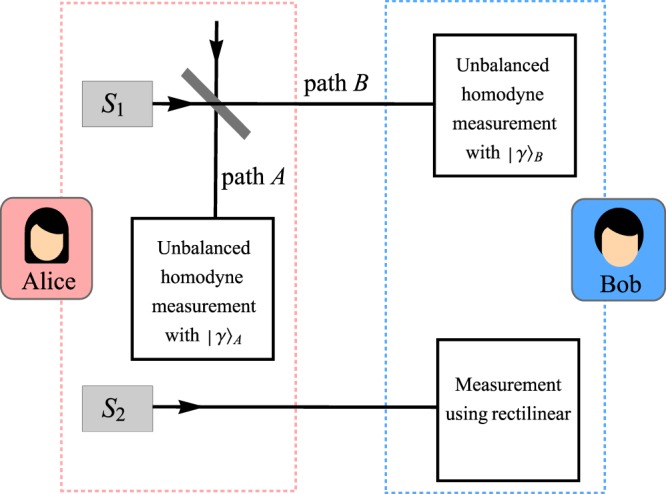
A schematic diagram of the proposed counterfactual protocol with *S*_1_ and *S*_2_ are the photon source for Setup 1 and Setup 2, respectively.

In this protocol, we assume that Alice and Bob share two setups as shown in Fig. 3. For definiteness, we named the setup which consists of source *S*_1_ as Setup 1 while the other as Setup 2.

In Setup 1, we will consider the protocol proposed in ref. ^12^, from this point onward will be referred to as SDI protocol (an acronym derived from its Single-photon entanglement nature in a Device Independent framework), which is based on single-photon entanglement protocol^13^. Setup 1 starts as Alice triggers the single photon source *S*_1_. The resulting state from single photon incident on the 50:50 beam splitter (BS) is given by

10 $$| \Psi \rangle =\frac{1}{\sqrt{2}}({| 1\rangle }_{A}{| 0\rangle }_{B}-{| 0\rangle }_{A}{| 1\rangle }_{B})$$

where *A* and *B* are the path towards Alice and Bob, respectively. Both parties then commit to an unbalanced homodyne measurement with a strong coherent state ${| \gamma \rangle }_{j}$ where *j* ∈ {*A*, *B*} represent the coherent state used in Alice’s and Bob’s measurement setting, respectively. The event of either Alice’s or Bob’s detector click corresponds to the binary value for the bit strings.

A certain amount of secrecy (i.e. non zero value for Eve’s uncertainty) is assured when Alice’s and Bob’s measurements are tested for violation of the Clauser-Horne (CH) inequality^14^:

11 $${I}_{CH}={Q}_{AB}(0,0,\rho )+{Q}_{AB}(s,0,\rho )+{Q}_{AB}(0,-s,\rho )-{Q}_{AB}(s,-s,\rho )-{Q}_{A}(0,\rho )-{Q}_{B}(0,\rho )$$

where *Q*_*AB*_(*α*, *β*, *ρ*) is the joint probability distribution of the absence of photon(s) events in Alice’s and Bob’s detectors given as

12 $${Q}_{AB}(\alpha ,\beta ,\rho )=\,{\rm{t}}{\rm{r}}\,({\widehat{Q}}_{A}(\alpha )\otimes {\widehat{Q}}_{B}(\beta )| \rho ).$$

The *α* and *β* are the coherent displacements of the path accessible to Alice and Bob, respectively. If Eq. (11) violates the inequality  −1 ≤ *I*_*CH*_ ≤ 0 then the secrecy is guaranteed as the nonlocality is satisfied. We refer the detail description of Setup 1 to SDI protocol.

Meanwhile, in Setup 2, Alice would prepare the qubit to be in either horizontally polarised state $| H\rangle$ or vertically polarised state $| V\rangle$. She would then submit this qubit to Bob where he will measure it in the rectilinear basis (this can be achieved by a polarising beam splitter with two detectors) and the measurements would distinguish between the polarisation states perfectly. For the sake of simplicity, we shall assume that the channel for Setup 2 is completely error free. This is not unreasonable as given the fact that the states can be distinguished perfectly, even by Eve, one can imagine that there is no reason for them to be transmitted as single photons subject to a depolarising channel; rather these states can be essentially ‘broadcast’ and the only real critical issue is to have it authenticated. Note that, we also do not put the requirement that both setups need to be performed simultaneously.

Now, using the results from both setups we can established the key as follows. We discard the result for all runs in Setup 2 that correspond to bit 1 in Setup 1. The remaining bits from Setup 2 will then serve as key strings for Alice and Bob. The protocol can now be outlined as follows.

1. Alice submits a photon to the 50:50 beam splitter, resulting in an entangled states of single photon and vacuum that is accessible to both Alice (in path *A*) and Bob (in path *B*).
2. Both of them would make a homodyne measurement, identical to the ones presented in SDI protocol.
3. After completing the transmission and measurement process, Alice and Bob would estimate the CH value on the measurement results and perform error correction procedure.
4. Note that steps 1 to 3 is identical to the SDI protocol, with the exception of privacy amplification which we do not execute.
5. Alice sends to Bob a string of polarised photon.
6. Bob measures the states using rectilinear basis.
7. Based on the results of step 2 and 5, the legitimate parties will discard the rounds in both setups which corresponds to bit 1 of Setup 1.
8. The remaining bits would then be used as a raw key.

It is worth noting that we are proposing an equivalent protocol to CQKD within a device independent scenario. By equivalence we mean that the protocol actually capitalises on the nature of single photon entanglement while the bits used for key is derive from the case where photons have not travelled to Bob but only to Alice. This is in fact the working principle for the CQKD. In what follows we will provide a heuristic analysis of the proposed protocol’s security.

### Security analysis

Supposedly, Alice and Bob share *N* bit strings in which we consider that on average, there would be an equal number between bit 0 and bit 1. Within these *N* bits, there are *U* bits that is unknown to Eve in which half of them will eventually be discarded. Hence, the possible ways for the parties to throw out the bits, *W* can be determine as follows

13 $$W=\frac{U!}{\frac{U}{2}!\left(U-\frac{U}{2}\right)!}$$

Eve’s uncertainty, *U*_*E*_ related to the unknown bits is given by the Shannon entropy as

14 $${U}_{E}={\log }_{2}W$$

Now, let us apply the above scenario in which Alice and Bob would initially share *N* bits string to the SDI protocol. Similarly, we imagine that Eve would not have any knowledge on *U* bits out of these *N* bits. Then, Eve’s uncertainty per bit for this protocol is given by

15 $$\frac{U}{N}\approx {p}_{NL}$$

which is approximately equal to the probability of Eve sending a nonlocal box, *p*_*NL*_.

Let us consider a scenario where Alice and Bob discard an equal fraction of bits in the SDI protocol i.e. the bits which correspond to Eve sending nonlocal boxes is halved. Therefore, the uncertainty that she has in that scenario would be

16 $${U}_{SDI}=\frac{U}{2}$$

We define *R* as the ratio of the uncertainty of this protocol to Eve’s uncertainty when half of SDI protocol bit are discarded written as

17 $$R=\frac{{\log }_{2}W}{\frac{U}{2}}$$

In the limit of long keys i.e. as *U* approaching infinite, we obtain

18 $$\mathop{\mathrm{lim}}\limits_{U\to \infty }R=2$$

This is of course the result that we would attain considering that the number of the two bits are equal.

With Eve’s uncertainty, *ε*_*u*_ = *p*_*NL*_ ⋅ *R* and Eve’s information *I*_*AE*_ = 1 − *ε*_*u*_, the key rate, *K* is given by the following formula

19 $$K=1-{I}_{AE}-h({e}_{AB})$$

in which *h*(*p*) = − *p*log_2_*p* − (1 − *p*)log_2_(1 −*p*) is the binary entropic function. Note that *e*_*AB*_ is the error between Alice and Bob, which corresponds to Setup 1. Hence, it is instructive to compare the performance of DI-CQKD with SDI protocol. We note that the key rate that is described in Eq. (19) should be divided by 2 when comparing the protocols. This is due to our assumption that the number of bit ‘0’ and ‘1’ in the string are necessarily the same.

**Figure 4:**
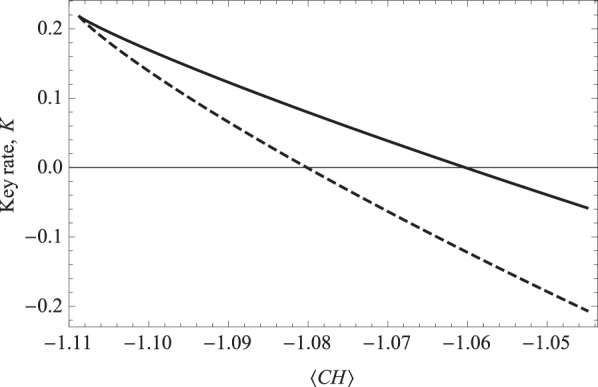
Key rate versus estimation of local violation, $\langle CH\rangle$. The dashed line represent the achievable key rate of SDI protocol, while the solid curve corresponds to the DI-CQKD protocol.

As we can see from Fig. 4, the maximum key rate achievable for the DI-CQKD protocol (represented as the solid curve) being approximately 0.22, which is the same as the SDI protocol described by the dashed curve. However, it is obvious from the graph that the key rate of DI-CQKD is non zero for a CH violation up to about  −1.06 whereas the SDI protocol obtain a non zero key rate only up till  −1.08 of the CH violation. The DI-CQKD perform better than the SDI protocol, as the key rate of DI-CQKD remains greater than the key rate of SDI protocol throughout the graph. This obvious increment is the result of Eve’s information being suppress in the DI-CQKD making her uncertainty per bit is twice than that of SDI protocol.

## Conclusion

In this work, we have outline the CQKD protocol as described by refs. ^2,11^ and analysed the security of the CQKD within a device independent context. We eventually show that the security of the protocol is compromised as the protocol is reproducible using separable states, resulting in an entirely classical correlations between the systems where the states can actually be predetermined by Eve. We further show that the need for the legitimate parties to disclose ‘which detector clicked’ in the public channel has given Eve access to the information of the shared key despite the state being entangled. This is because the entanglement is only between the first two qubits while the polarised state that is used to establish the key string is not. Hence, we propose a new (equivalent) version of CQKD within device independent scenario, the DI-CQKD, with the basic building block being the SDI protocol of the previous chapter.

We then compare the performance of DI-CQKD and SDI protocol. Based on our findings, both the DI-CQKD protocol and SDI protocol achieve the same highest key rate of approximately 0.22. However, we note that the performance of DI-CQKD protocol exceeds SDI as a positive key rate is obtained for a violation of CH up till  −1.06 compared to SDI that is only up to  −1.08 with the DI-CQKD key rate being greater than that of SDI the entire time.

Finally we show how one can actually use this equivalent protocol to construct a DI-CQKD where selected runs of a conventional CQKD^11^ is randomly substituted with runs to determine a Bell violation.
